# Causes of Blindness among Adult Yemenis: A Hospital-based Study

**DOI:** 10.4103/0974-9233.53367

**Published:** 2008

**Authors:** Saleh A. Al-Akily, Mahfouth A. Bamashmus

**Affiliations:** 1 From Eye Department, Faculty of Medicine and Health Sciences, Sana'a University, Sana'a, Republic of Yemen; 2 From Ibn Al-Haitham Eye Center, University of Science and Technology, Sana'a, Republic of Yemen

**Keywords:** blindness, cataract, glaucoma, corneal opacity, Yemen

## Abstract

**Purpose:**

This hospital-based retrospective study was aimed to assess the causes of blindness among adults aged 17 years and over who attended a teaching eye hospital in Yemen.

**Methods:**

The case notes of 3845 consecutive new patients over 12 months attending Ibn Al-Haitham Eye Center which is affiliated to the University of Science and Technology in Sana'a (the capital of Yemen) were retrieved and analysed. Data collected included age, gender, chief complaint and complete eye examination.

**Results:**

7.7 percent (296) were uniocularly blind and 11.2 percent (432) were binocularly blind (best corrected visual acuity <3/60 in the better eye). The leading causes of uniocular blindness were cataract, trauma related ocular complications, corneal opacity, amblyopia and glaucoma. Binocular blindness was mainly due to cataract, glaucoma, diabetic retinopathy, age related macular degeneration and corneal opacity.

**Conclusions:**

These data imply that the preliminary results give us some insight about the magnitude of the problem of blindness in Yemen while awaiting a national survey on the prevalence and causes of blindness. Cataract was found to be the main cause of unilateral and bilateral blindness and this will require surgical relief, either in public hospitals, private hospitals and clinics, or in eye camps. Trauma related ocular complications were found to be the second most common cause of uniocular blindness. Health education, implementing work safety measures and bringing ophthalmological care to the doorstep of underprivileged rural community will improve their level of awareness.

The Republic of Yemen is a Middle Eastern developing country lies in the Arabian Peninsula, southwest of Asia. Bordered on the north by Saudi Arabia, on the south by the Arabian Sea and the Gulf of Aden, on the east by the Sultanate of Oman and on the west by the red sea. Its total area exceeds 555,000 km excluding the Empty Quarter Dessert. The total population of Yemen is 19,685,161 (49.5 percent of whom are aged 17 years and above). The capital city, Sana'a has an estimated population of 1,488,108.^1^ The country is poor with a human development index of 0.486; human poverty index is 47.0, a life expectancy of 61.1 and a literacy rate of 50 percent.^2^

Uniocular blindness is defined as a corrected visual acuity 3/60 or below in one eye. Binocular blindness is defined as a corrected visual acuity of 3/60 or below in both eyes.^3^ Blindness constitutes significant public health problem in many countries.

Family health survey conducted by Ministry of Health in 2003 revealed that 2.9 percent of the population had some sort of self reported handicap, with visual handicap representing 36 percent of the total identified disabled individuals in the country.^4^

The available data suggests that in the year 2000 there were approximately 50 million blind people in the world. The majority live in Asia and Africa. Approximately 8–10 million people become blind each year and it is estimated that around 6–8 million people who are blind die each year. The result is a net increase of 1–2 million blind person per year. The increase in blindness is due to an increasing world population and increased life expectancy, with more people in the world leaving beyond the age of 60 years.^5^

Of the 50 million blind people in the world, it is estimated that, approximately half is due to cataract, 15 percent due to trachoma and 4 percent due to paediatric blindness. The other causes of blindness are glaucoma (approximately 15 percent) and diabetic retinopathy (5 percent).^5^ Despite the efforts of UN agencies, national governments and non-governmental organizations, blindness is an increasing problem causing loss of quality of life to the individual and an economic burden on the individual, family and society in general.

There has been no national blindness survey in Yemen. The available data on the prevalence and causes of blindness in Yemen are studies done in focal areas of the country.^6^,^7^ To our knowledge there has been no published hospital-based studies on the causes of blindness in Yemen.

## Patients and Methods

We studied 3845 consecutive patient records (2246 males and 1599 females) who attended the Ibn Al-Haitham Eye Centre in Sana'a during the academic year 2001/2002. The study was restricted to persons aged 17 years and over. Children were not included.

After initial registration and history taking, all patients had their visual acuity measured using the Snellen visual acuity chart projector at 6 meters; spectacles were worn if necessary to give the best corrected vision. Patients were then examined with a slit lamp, direct or indirect ophthalmoscopy, the intraocular pressure was measured by Goldmann applanation tonometer in the suspected cases of glaucoma and to those above the age of 40 years. Those patients with a best corrected visual acuity of less than 3/60 in both eyes were categorized as binocularly blind (WHO standards) and those with best corrected visual acuity of less than 3/60 in one eye but 3/60 or better in the other eye were classified as uniocularly blind.

In most cases the cause of blindness was a single disorder. When there was more than one pathology in a patient, the WHO recommendation was adhered to: namely the most avoidable or preventable pathology was chosen or, alternatively, the cause that led to the last event rendering the individual sightless^8^. This principle was also adhered to while classifying the causes of blindness in unilateral cases when multiple causes were present in one eye. After establishing the diagnosis, medicines or glasses were prescribed as appropriate. Data were entered into an Excel 2000 program and analyzed.

## Results

A total of 3845 new patients aged over 17 years were seen during the study period. Of these, 296 (7.7 percent) were uniocularly blind while 432 (11.2 percent) had binocular blindness.

Out of the 296 uniocular blind there were 196 (66.2 percent) males and 100 (33.8 percent) females. Cataract and ocular trauma related complications were the commonest causes of uniocular blindness. Table 1 shows the major causes of uniocular blindness.

**Table 1:** Causes of Unilateral Blindness

	Male	Female	Total	%
Cataract	38	25	63	21.3

Trauma Complications	50	10	60	20.3

Corneal Leucoma (microbial & dystrophy)	21	13	34	11.5

Amblyopia	18	14	32	10.8

Glaucoma	13	7	20	6.8

Retinal Detachment	12	4	16	5.4

Diabetic Retinopathy	8	0	8	2.7

Others	34	29	63	21.3

Total	196	100	296	100.0

Table 2 shows the major causes of bilateral blindness. Once again cataract was the commonest cause of bilateral blindness. There were 258 (59.7 percent) males and 174 females (40.3 percent). Table 3 shows the statistical analysis of bilateral blindness in males and females. Table 4 compares leading causes of bilateral blindness in selected countries.

**Table 2:** Causes of Bilateral Blindness

	Male	Female	Total	%
Cataract	112	88	200	46.3

Glaucoma	32	13	45	10.4

Diabetic Retinopathy	23	14	37	8.6

ARMD	25	10	35	8.1

Corneal Leucoma (microbial & dystrophy)	16	11	27	6.3

Uncorrected Aphakia	8	11	19	4.4

Optic Atrophy	9	5	14	3.2

Degenerative Myopia	3	9	12	2.8

Trauma	7	2	9	2.1

Keratoconus	6	1	7	1.6

Retinitis Pigmentosa	6	1	7	1.6

Uveitis	2	3	5	1.2

Retinal Detachment	3	1	4	0.9

Others	6	5	11	2.5

Total	258	174	432	100.0

**Table 3:** Causes of Bilateral Blindness with Statistical Analysis

Disease	Male	Female	Odds Ratio (Cl)	Risk Ratio (Cl)	Chi-square	P-value	Significance
Cataract	112	88	0 .75 [0.51; 1.10]	0.86 [0.70; 1.05]	1.87	0.172	Not Significant

Glaucoma	32	13	1.75 [0.89; 3.45]	1.66 [0.90; 3.07]	2.21	0.137	Not Significant

Diabetic Retinopathy	23	14	1.12 [0.56; 2.24]	1.11 [0.59; 2.09]	0.02	0.888	Significant

ARMD	25	10	1.76 [0.82; 3.76]	1.69 [0.83; 3.42]	1.67	0.196	Not Significant

Corneal Leucoma (microbial & dystrophy)	16	11	0.98 [0.44; 2.17]	0.98 [0.47; 2.06]	0.02	0.879	Significant

Optic Atrophy	8	11	0.47 [0.19; 1.20]	0.49 [0.20; 1.19]	1.86	0.173	Not Significant

Degenerative Myopia	9	5	1.22 [0.40; 3.71]	1.21 [0.41; 3.56]	0.01	0.939	Significant

Trauma	3	9	0.22 [0.06; 0.81]	0.22 [0.06; 0.82]	4.79	0.029	Significant

Keratoconus	7	2	2.40 [0.49; 11.68]	2.36 [0.50; 1.23]	0.6	0.440	Not Significant

Retinitis Pigmentosa	6	1	4.12 [0.49; 34.52]	4.05 [0.49; 33.32]	1.05	0.305	Not Significant

Uveitis	6	1	4.12 [0.49; 34.52]	4.05 [0.49; 33.32]	1.05	0.305	Not Significant

Retinal Detachment	2	3	0.45 [0.07; 2.69]	0.45 [0.08; 2.66]	0.2	0.656	Not Significant

**Table 4:** Comparison of the Leading Causes of Bilateral Blindness in Selected Countries

Country	1st cause	2nd cause	3rd cause	4th cause
Yemen	Cataract	Glaucoma	Dlabetic retinopathy	Age-related macular degeneration

Jordan	Cataract	Diabetic retinopathy	Glaucoma	Comeal opacity

Egypt	Corneal Opacity	Cataract	Glaucoma	Optic nerve atrophy

India	Cataract	Glaucoma	Diabetic retinopathy	Corneal Opacity

Scotland	Age-related macular degeneration	Glaucoma	Cataract	Diabetic retinopathy

Source: Reference # 13, 18, 19, 20

## Discussion

The results of a hospital-based survey are not necessarily representative of the total population with eye disease; this is because the patients attending to the hospital are self-selecting. However, it can provide us with some perspective on pattern of eye diseases seen in the hospital relative to the total blind population.

We did not study cases of low vision and restricted our study to blindness, because we felt that patients with low vision may not seek medical advice while blind patients most probably will. This may make our study more nearly representative of the entire population of Yemen. Although this study cannot be taken as indicative of the whole situation in Yemen it tells us on the relative frequency of causes of blindness.

### Cataract:

Cataract was found to be the main cause of uniocular (21.3 percent) and binocular blindness (46.3 percent) in Yemen which is a developing country that need to expand further medical and surgical facilities especially in rural area; this is similar to that found in other studies in developing countries.^3^,^5^ where it contributes to 57.7 percent of cases of blindness in India and Latin America, and was responsible for 45.2 percent in the Middle East crescent.^8^ In Yemen as in other developing countries there is a common believe among most elderly that reduced vision is considered a normal aging process.

### Glaucoma:

Glaucoma was the fifth most common cause of unilateral blindness (6.8 percent) and the second most common cause of bilateral blindness (10.4 percent). The prevalence of glaucoma in our population has not been studied but in a study conducted in a major hospital in Sana'a it was noticed that patients with glaucoma tend to present at a late stage as there is no screening program.^9^ Many of the glaucoma patients presented very late with severe visual loss and typical disc changes hence, visual field assessment was not essential in reaching diagnosis in all cases.

### Diabetic Retinopathy:

Diabetic retinopathy is the third cause of bilateral blindness (8.6 percent) and this is almost similar to other studies' figures in India.^5^ Most studies in developing countries show that diabetic retinopathy is one of the leading causes of blindness preceded by cataract and glaucoma.^10^

Blindness caused by diabetic retinopathy has been decreasing worldwide since the introduction of laser photocoagulation and advances in vitreoretinal surgeries.^11^ Vitreoretinal services is not well established in most developing countries and if available, it is expensive to the majority of patients. Late presentations of diabetic retinopathy patients still hang on to a concept between Yemeni patients that they may loose vision if they receive laser treatment early. Other patients who receive laser treatment think they have become immune to further progression of the diabetic retinopathy and start neglecting the control of their diabetes.^12^

### Age Related Macular Degeneration:

Age related macular degeneration (ARMD) is the leading cause of blindness in western countries.^13^ This is not the case in our study and in neighboring countries where it was found to be the fourth cause of bilateral blindness (8.1 percent). Results from community based survey revealed a high prevalence of ARMD (in age 50 years and above) as a cause of blindness (14.3 percent).^6^ Possible explanations are the lower life expectancy of our population compared to Western countries, and the high prevalence of illiteracy among the elderly who do not appreciate loss of central vision needed for reading.

### Corneal Opacity:

The importance of corneal opacification as a cause of blindness varies. It depends on the triggering factors for opacification in any individual.^14^ In our study we included opacities caused by bacterial and viral infections, dystrophies, those complicating trichiasis and entropion or other causes apart from ocular injuries and keratoconus. Corneal opacities was found to be the third most common cause of unilateral blindness (11.5 percent) and the fifth most common cause in bilateral blindness (6.3 percent). Corneal donations are not available in Yemen and there is a long waiting list of patients in need of corneal grafts.

### Trauma:

Trauma is a common cause of blindness in developing countries. In Yemen trauma was found to be high compared to some published studies in neighboring countries and that is related to stone and stick related injuries, road traffic accidents, landmines, bomb explosions and fireworks.^17^ In our study trauma related ocular complications was found to be the second most common cause of uniocular blindness responsible for 20.2 percent. In Jordan it was responsible for 11 percent,^10^ while in Lebanon was responsible for 5 percent^15^ and in Cambodia was responsible for 13 percent.^16^ Unfortunately there is no corneal and vitreoretinal subspecialty services at the time of conducting this study leading to most cases having corneal and posterior segment lesions that ended with blindness.
